# How Do Acquired Political Identities Influence Our Neural Processing toward Others within the Context of a Trust Game?

**DOI:** 10.3389/fnhum.2018.00023

**Published:** 2018-02-02

**Authors:** Chien-Te Wu, Yang-Teng Fan, Ye-Rong Du, Tien-Tun Yang, Ho-Ling Liu, Nai-Shing Yen, Shu-Heng Chen, Ray-May Hsung

**Affiliations:** ^1^School of Occupational Therapy, College of Medicine, National Taiwan University, Taipei, Taiwan; ^2^Department of Psychiatry, National Taiwan University Hospital, Taipei, Taiwan; ^3^Department of Sociology, National Chengchi University, Taipei, Taiwan; ^4^Department of Biological Science and Technology, National Chiao Tung University, Hsinchu, Taiwan; ^5^Department of Economics, National Chengchi University, Taipei, Taiwan; ^6^Regional Development Research Center, Taiwan Institute of Economic Research, Taipei, Taiwan; ^7^Department of Imaging Physics, University of Texas MD Anderson Cancer Center, Houston, TX, United States; ^8^Department of Psychology, National Chengchi University, Taipei, Taiwan; ^9^Research Center for Mind, Brain, and Learning, National Chengchi University, Taipei, Taiwan

**Keywords:** social identity, trust, political orientations, decision making, neuroeconomics

## Abstract

Trust is essential for mutually beneficial human interactions in economic exchange and politics and people’s social identities notably have dramatic effects on trust behaviors toward others. Previous literature concerning social identities generally suggests that people tend to show in-group favoritism toward members who share the same identity. However, how our brains process signals of identity while facing uncertain situations in interpersonal interactions remains largely unclear. To address this issue, we performed an fMRI experiment with 54 healthy adults who belonged to two identity groups of opposing political orientations. The identity information of participants was extracted from a large-scale social survey on the 2012 Taiwan presidential election. Accordingly, participants were categorized as either the Kuomintang (KMT) or the Democratic Progressive Party (DPP) supporters. During the experiment, participants played trust games with computer agents with labels of the same or the opposing political identity. Interestingly, our results suggest that the behaviors of the two groups cannot be equally attributed to in-group favoritism. Behaviorally, only the DPP supporter group showed a significant trust preference toward in-group members, which did not hold for the KMT supporter group. Consistently, neurophysiological findings further revealed that only the DPP supporter group showed neuronal responses to both unexpected negative feedback from in-group members in anterior insula, temporoparietal junction, and dorsal lateral prefrontal cortex, as well as to unexpected rewards from out-group members in caudate. These findings therefore suggest that acquired identities play a more complex role in modulating people’s social expectation in interpersonal trust behaviors under identity-relevant contexts.

## Introduction

Trust is one of the most socially sophisticated skills that are essential for mutually beneficial human interactions (Fett et al., 2014), such as in economic exchange and political dealmaking (Kosfeld et al., 2005). Moreover, trust is specifically important when facing uncertain situations (Brann and Foddy, 1987). Social identities, which reflect a person’s shared characteristics and/or values associated with the group s/he belongs to (Tajfel and Turner, 1979), seems to be capable of affecting trust behaviors toward others (Huettel and Kranton, 2012). For example, when individuals recognize each other as coming from the same social group, both the interpersonal trust and trustworthiness increase; conversely, if the investors and the trustees came from different groups, levels of trust and trustworthiness decline (Stanley et al., 2012).

The social identity theory of intergroup behavior (Tajfel and Turner, 1986) provides a plausible model to explain the effect of social identity on people’s decision making. The theory posits that individuals strive to maintain a positive perception of their in-groups (i.e., with the same identity) while showing negative orientations toward their out-groups (i.e., with a different identity). Previous empirical evidence has revealed that individuals who have strong senses of identity to their group are more likely to have a stronger sense of group commitment (Huddy, 2003) and to discriminate against an out-group individual when they were requested to distribute resources between in-groups and out-groups (Perreault and Bourhis, 1999; Chen and Li, 2009; Gummerum et al., 2009). Such identity-based in-group/out-group interaction has been linked to both social cognitive (e.g., mentalizing) (Freeman et al., 2010) and affective processes (Cikara and Van Bavel, 2014). For example, people seem to engage more deliberate mentalizing processes when splitting an endowment with an out-group member than with an in-group member in a dictator game (Telzer et al., 2015). Psychologically, people tend to create a tendency of outcome expectation regarding in-group versus out-group interactions (Harris and Fiske, 2010), likely based on past experiences and knowledge (Olson et al., 1996). When in-group members behave in ways that violate rather than comply with socially stereotypical expectancies, perceivers tend to experience greater affective disturbance and involve more cognitive processing to resolve the unexpected uncertainty (Jost et al., 2014).

It seems to be difficult, however, to directly generalize the prediction of the social identity theory to acquired identities (e.g., Huddy, 2001, 2003). The theory mainly originated from empirical evidence with assigned identities that lack the identity choice freedom for people, a critical characteristic that is fundamentally different from acquired identities. Specifically, the group cohesion induced by acquired identities in which members have the freedom to voluntarily choose their groups could be qualitatively different from that induced by assigned identities in which members had no such freedom (Turner et al., 1984). In the context of trust-based interaction, in-group favoritism seems to occur only when participants believe other in-group members will reciprocate the favor (Yamagishi and Kiyonari, 2000). However, a few of recent studies indeed found significant differences in trust patterns between different political identity groups which cannot be fully explained by in-group favoritism alone (e.g., Carlin and Love, 2013; Yang et al., 2014; Hernandez and Minor, 2015). These data altogether suggest that shared contents and structures of beliefs about trust within a group can be critical in shaping group members’ trust behaviors (Yamagishi, 2003).

Although quite a few studies have investigated the distinct neural mechanisms underlying trust reciprocity and feedback learning (Delgado et al., 2005; King-Casas et al., 2005; Baumgartner et al., 2008; Krueger et al., 2008; Chang et al., 2011; Kang et al., 2011; Aimone et al., 2014; Fareri et al., 2015; Bellucci et al., 2017), the modulatory nature of social identity upon trust behaviors and its neurobiological underpinnings remains relatively unclear. Typically, negative feedback that violate pre-existing social expectations during economic decisions (e.g., from trustees with close social distance) results in increased activation in regions implicated in affective processing [anterior insula (AI) and amygdala], mental inferences [temporoparietal junction (TPJ)], and cognitive control [dorsolateral prefrontal cortex (DLPFC) and anterior cingulate cortex (ACC)] (Aimone and Houser, 2011; Cloutier et al., 2011; Aimone et al., 2014), whereas positive feedback that violate pre-existing social expectations (e.g., from trustees with bad reputation) results in increased activation in reward-related regions (caudate) (Delgado et al., 2005). It has been recently shown that some ascribed social identities (e.g., age, sex, and ethnicity) could modulate activations during trust-related interaction within brain regions including caudate/striatum, AI, amygdala, TPJ, ACC, and DLPFC (Riedl and Javor, 2012; Stanley et al., 2012). These regions were known to be involved in reward learning, emotional awareness, mentalizing, and social evaluation (Riedl and Javor, 2012). Up to date, however, how our brains process signals of acquired identities (e.g., political orientations in this case) while facing uncertain situations under the contexts of interpersonal trust remains largely unclear.

**FIGURE 1:**
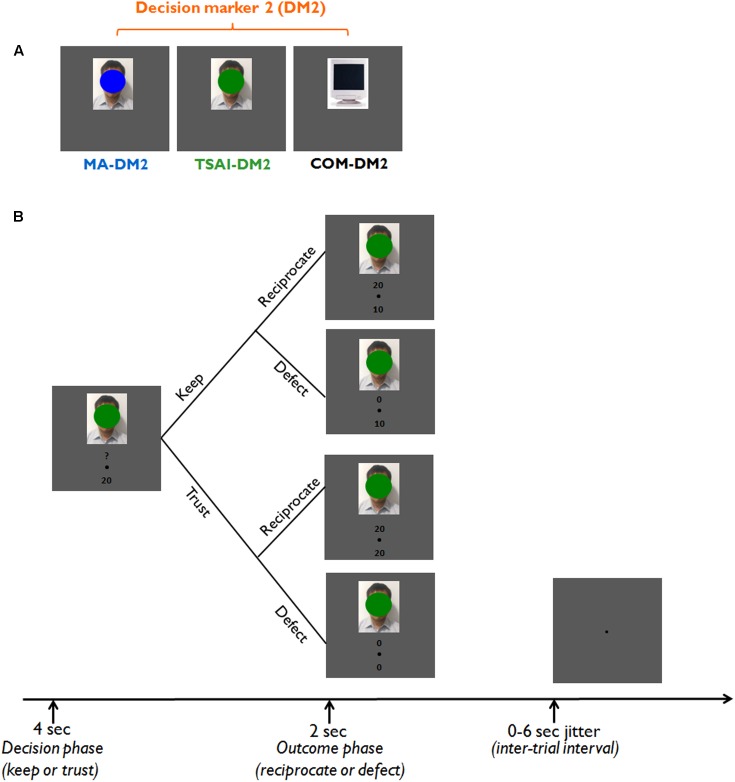
Experimental design. **(A)** Different types of partners (decision marker 2, DM2). For each trial, participants (Decision Maker 1, DM1) could play the trust game with one of the three different types of partners (DM2). A DM2 could be a face covered by a blue patch (MA-DM2, representing a DM2 who voted for Ma in the 2012 presidential election of Taiwan), a face covered by a green patch (Tsai-DM2, representing a DM2 who voted for Tsai in the 2012 presidential election of Taiwan), or a computer (representing a neutral DM2). Note that the face stimulus was equally likely to be a male or a female face. **(B)** A typical trial sequence of the trust game. Each trial started with a 4-s decision phase (an image of one type of DM2) and a 2-s outcome phase in which we presented the gain of that trial and the response from the corresponding DM2 had participants chosen to trust.

To address this question, we performed a well-validated fMRI trust game paradigm (Phan et al., 2010) in which participants were required to play as an investor (Decision Maker 1, DM1) in a binary trust game with different simulated partners (Decision Maker 2, DM2) who have either the same or an opposing political identity as DM1 (**Figure 1A**). In each trial of the trust game, participants (DM1) were required to decide to either trust the corresponding DM2 by sending the initial endowment to her/him/it or distrust the corresponding DM2 by keeping the initial endowment. The response of any DM2 could either be to reciprocate or to defect in the case that DM1 decided to trust (**Figure 1B**). Behaviorally, according to the social identity theory which indicates that in-group favoritism is pervasive for any group with an established identity, we hypothesized that all participants would show higher trust rates when they were playing with their trustees having the same political identity than with their trustees having different political identity. Neurophysiologically, we hypothesized that political identity would modulate the neural activation patterns involved in reward outcome, emotional awareness, mentalizing, and social evaluation, including the caudate/striatum, AI, amygdala, ACC, TPJ, and DLPFC. In particular, we expected that activations within the neural network subserving affective and cognitive processing would vary with different conflicts of social expectancies arising from playing with partners of the same or different political identity.

## Materials and Methods

### Participants

All participants in the current fMRI experiment were actually enrolled to participate in a three-stage research project and the current fMRI experiment was the third stage. Initially in the first stage, 1550 individuals participated in a large-scale sociological survey concerning their political orientation immediately after the Taiwan presidential election in 2012. Accordingly, participants were categorized into two groups according to whether they voted for the KMT candidate Ying-Jeou Ma or for DPP candidate Ing-Wen Tsai. The tension between supporters of the two political parties has been notoriously high during important elections (e.g., president, mayors, legislators) for the past two decades, most likely induced by opposition on the issue of Taiwan’s independence during the presidential elections (please see Supplementary Materials for more details). Note that the major divergence between two parties in the presidential election of 2012 was mainly on the issue of national identity (Cheng, 2013; Liu and Li, 2017).

In the second stage, 208 out of 1550 participants who had completed the first stage survey were recruited to perform a behavioral economic study about political identities and trust (Yang et al., 2014). Due to the occurrence of experimental errors, we discarded 16 participants in this stage. The remaining 192 participants underwent three rounds of repeated trust games with one player for each round. Specifically, they played games with a random player in the first round, with a player showing his/her political identity in the second round, and with a player whose political identity matched participants’ free choice.

For the current third-stage fMRI experiment, we recruited 58 right-handed participants out of the 192 healthy adults from the second-stage experiment (34 females, mean age of 23.76 ± 3.29 y/o, average education of 15.37 years) to participate the current fMRI study. Data from four individuals were excluded due to serious motion artifacts. Among the 54 participants, half of them voted for the KMT candidate Ying-Jeou Ma (16 females, mean age of 23.30 ± 0.67 y/o) and the other half of them voted for the DPP candidate Ing-Wen Tsai (18 females, mean age of 24.22 ± 4.60 y/o) in the 2012 Taiwan presidential election. None of them had a history of neurological or psychiatric disorders, or were taking psychotropic medications at the time of the study. The two groups showed no significant difference in terms of age (*p* = 0.31), gender ratio (*p* = 0.57), and years of education (*p* = 0.22). Note that all fMRI data were collected from April to November 2014, which was roughly 2 years after the 2012 presidential election.

The experimental protocol has been reviewed and approved by the Institutional Review Board at National Taiwan University (201210HS031), and all participants provided signed informed consent before the experiment. All experiments were performed in accordance with relevant guidelines and regulations for protecting human participants.

### General Procedures

Before fMRI scanning, all participants underwent assessments of the Big Five personality traits (Goldberg, 1990) and social capital (Lin, 2001), and completed 12 practice trust game trials on a laptop in a quiet room. After fMRI scanning, participants rated their general subjective unpleasantness when receiving outcome feedback from trustee players (i.e., reciprocate or defect) in the trust game. We used a nine-point Likert scale, where one indicated “extremely unhappy (utterly depressed, completely down)” and nine indicated “extremely happy (feeling ecstatic, joyous)” (Yang et al., 2014).

#### Trust Game

We used a binary trust game paradigm to investigate participants’ trust behaviors. A computerized trust game was chosen as a means for having people interact with someone strictly based on political identity. The computer program allowed the experimenters to control the stimuli that the participants would see (i.e., group membership), as well as randomize both trust and reciprocity trials. The whole experiment sequences were coded with the Matlab-based Psychophysics Toolbox (Brainard, 1997; Pelli, 1997).

For each trial of the trust game, participants received a starting fund of 20 monetary units (MU) and played as an investor (DM1) which requires them to decide whether to invest the 20 MU to the trustee player agent (Decision Maker 2, DM2) in that trial. If a participant chooses to hold the investment (KEEP), then the trial is finished with an outcome in which the starting fund is equally divided between DM1 and the corresponding DM2 (i.e., with each one receives 10 MU). If a participant chooses to invest (TRUST), then the starting fund doubles to 40 MU and the outcome is to be decided by DM2 between the following two scenarios (Supplementary Table S1): (1) RECIPROCATE: to split the money equally with the participant so that each receives 20 MU and (2) DEFECT: DM2 keeps the entire amount of 40 MU and DM1 receives 0 MU.

Participants were informed that they would play multiple one-shot games with three different types of computer opponents. The humanoid computer players will be displayed as face images whose facial features were mostly covered by either a blue oval representing those who voted the KMT candidate Ying-Jeou Ma in the 2012 Taiwan presidential election (MA-DM2), a green oval representing those who voted the DPP candidate Yin-Wen Tsai (TSAI-DM2) in the 2012 Taiwan presidential election or simply a computer monitor image (COM-DM2). The blue and green colors were used because they are the colors for the KMT and DPP emblem, respectively. The color patch coverage was applied to reduce any potential confounds from facial attractiveness, emotional expressions, etc. (please note that the face stimuli was equally probable to be a male or a female face). At the start of each trial, the image of DM2 for a given trial appeared for 4 s during which participants were instructed to make their choice by button press. Participants were only required to make a decision within the 4 s period (not a speeded decision), and thus we did not record or analyze the reaction times for each trial. However, we note that such design may limit our capability to investigate potential speed-accuracy trade-offs during the decision processes. The outcomes were provided immediately after this decision period by a DM2 image reappearing for 2 s along with information about the agent’s decision. This information was represented below the DM2 image as the amount of money sent back to DM1.

All participants were informed that the overall reciprocate rate across all DM2 types (MA-DM2, TSAI-DM2, and COM-DM2) was 50% (please see Supplementary Materials for detailed task instructions). In other words, participants only knew that half of DM2 would decide to reciprocate if participants decided to trust DM2 in a given trial. Participants had no further information regarding the corresponding decision pattern for any specific DM2 type, which formulates a context of uncertainty that is critical in studying trust behavior (Phan et al., 2010). In the current case, we can investigate the effect of identities without any explicit cued instruction for their political beliefs. Unbeknownst to all participants, however, we set a 50% reciprocate rate for all types of DM2. This design allowed us to focus our analysis on the effect of outcomes as the number of trials for the RECIPROCATE or DEFECT outcome would be the same (i.e., providing equal statistical power between participants for each condition).

We inserted an inter-trial interval (0–6 s jitter) with a fixation dot on a gray blank screen between trials (Ollinger et al., 2001a,b) and trial sequence was presented using a first-order counterbalanced design so that each type was preceded with similar percentage of other trial types (Weissman et al., 2008). Each participant performed six runs with each run composed of 12 trials with MA-DM2, 12 trials with TSAI-DM2, and 12 trials with COM-DM2, trial order randomized. In other words, they played with a total of 72 “DM2” for each type. At the end of the whole experiment, each participant was paid with a bonus according to the actual outcome summation from the trust game [the average outcome = 325.28 New Taiwan Dollar (NTD), standard deviation = 18.61 NTD, range = 299–438 NTD] in addition to a fixed payment for their participation. With respect to the participant’s political identity, there were a total of six conditions with different identity/outcome combinations:

(1) Same/Reciprocate (SR): In-group trials, participants were playing with a DM2 who has the same political identity (SAME-DM2) and received a positive feedback.
(2) Same/Defect (SD): In-group trials, participants were playing with a DM2 who has the same political identity and received a negative feedback.
(3) Different/Reciprocate (DR): Out-group trials, participants were playing with a DM2 who has different political identity (DIFF-DM2) and received a positive feedback.
(4) Different/Defect (DD): Out-group trials, participants were playing with a DM2 who has different political identity (DIFF-DM2) and received a negative feedback.
(5) Computer/Reciprocate (CR): Non-human control trials, participants were playing with a non-humanoid computer (COM-DM2) and received a positive feedback.
(6) Computer/Defect (CD): Non-human control trials, participants were playing with a non-humanoid computer (COM-DM2) and received a negative feedback.

### MR Data Acquisition

Structural and functional MRI imaging data were acquired on a 3T MR scanner (Skyra; Siemens, Erlangen, Germany). Tight but comfortable foam padding was used to minimize head motion, and ear plugs were used to reduce scanner noise. The scanning protocol included the acquisition of structural MR images (MPRAGE sequence, FoV = 256 × 256 mm, matrix = 256 × 256, TR = 2530 ms, TE = 3.3 ms, flip angle = 7°, slice thickness = 1 mm, no gap) and functional images [gradient echo-planar imaging (EPI) sequence, FoV = 220 × 220 mm, matrix = 64 × 64, TR = 2000 ms, TE = 30 ms, flip angle = 90°, 36 slices/slab covering the whole brain, slice thickness = 4 mm, no gap]. For each functional run, a total of 187 EPI volume images were acquired with oblique axial slices parallel to the AC–PC line.

### fMRI Image Preprocessing and Analysis

SPM8 (Wellcome Department of Imaging Neuroscience, London, United Kingdom) was used for image processing. The first four volumes of each functional session were discarded to allow for T1 equilibration effects. The remaining images underwent preprocessing, including reorientation, slice-timing correction, head motion correction, normalization to the EPI template with a resampled voxel size of 2 × 2 × 2 mm, and smoothing with an isotropic 10-mm full-width half-maximum (FWHM) Gaussian kernel. A two-stage general linear model was used to examine the effect sizes of each condition and to compare them at group level. Six regressors (corresponding to the six described trial types) were employed to model the onset of outcome presentation. At the first level analyses, a voxel-by-voxel multiple regression analysis of expected BOLD response for all six identity/outcome conditions was performed based on canonical hemodynamic response function models for each participant. Linear contrasts were applied to the obtained parameter estimates. Then, each effect of interest was tested across all participants. The data were high-pass filtered with a frequency cut-off at 128 s. At the second level analyses, we performed an ANOVA upon condition contrasts with two within-subject factors: DM2-TYPE (Same, Different, or Computer) and OUTCOME (Reciprocate vs. Defect). We used a corrected statistical threshold of *p* < 0.05 achieved by a voxel-wise *p* < 0.001 and an extent threshold of *k* > 70 voxels [using a cluster extent thresholding algorithm developed by Slotnick et al. (2003)] for all whole brain analyses. However, with the corrected statistical threshold, we did not observe a significant three-way interaction (DM1-GROUP × DM2-TYPE × OUTCOME). Given our primary research interest, we therefore investigated the three-way interaction effect with a less stringent threshold setting with uncorrected *p* < 0.001 and an extent threshold of *k* > 10 voxels for exploratory whole brain analyses. The reason for the use of less-stringent threshold settings is that the current design was trying to study a relatively complicated social interaction process, and due to scanning time constraints, we could only include a relatively limited number of trials for each condition. We therefore think that it would be helpful to provide the reader with exploratory results for the whole brain three-way interaction effect. Activations were overlaid on a high-resolution structural T1-weighted image template from SPM8, co-registered to the Montreal Neurological Institute (MNI) space.

#### ROI Analysis

To further dig into the activation patterns of the three-way interaction effect of DM1-GROUP × DM2-TYPE × OUTCOME within brain regions that have been reported in previous studies of trust-related behaviors, we performed region of interest (ROI) analyses. DLPFC, TPJ, AI, and caudate were identified individually through literature reviews. These regions were selected because of their role in trust decisions and social expectancy as reported by current literature (Cloutier et al., 2011; Huettel and Kranton, 2012; Riedl and Javor, 2012; Wardle et al., 2013; Aimone et al., 2014; Bellucci et al., 2017). Specifically, we extracted the following ROIs from the corresponding related literature as follows (a 6-mm radius sphere drawn from a designated coordinate): right AI [(42, 18, 4), Bellucci et al. (2017)], right TPJ [(50, -60, 34), Cloutier et al. (2011)], left TPJ [(-44, -58, 32), Cloutier et al. (2011)], right DLPFC [(10, 12, 52), Gabay et al. (2014)], and left caudate [(-10, 10, 16), Wardle et al. (2013)].

Region of interest analyses were carried out using extracted parameter estimates from selected ROIs for the SR, SD, DR, and DD conditions. These values were then used as input to a repeated-measures ANOVA. To further assess the relationship between the hemodynamic responses to political identity-eliciting stimuli and subjective unpleasantness to DM2s’ returns, multiple regression analyses were performed to determine which ROIs were significantly associated with unpleasantness ratings.

## Results

### Behaviors

There was no significant difference between two DM1 groups (MA-DM1 vs. TSAI-DM1) with respect to personality traits (*p* = 0.87) and measurements of social capital (*p* = 0.27). These data indicated that MA-DM1 and TSAI-DM1 group members had comparable psychological personality profiles and comparable social capital.

**FIGURE 2:**
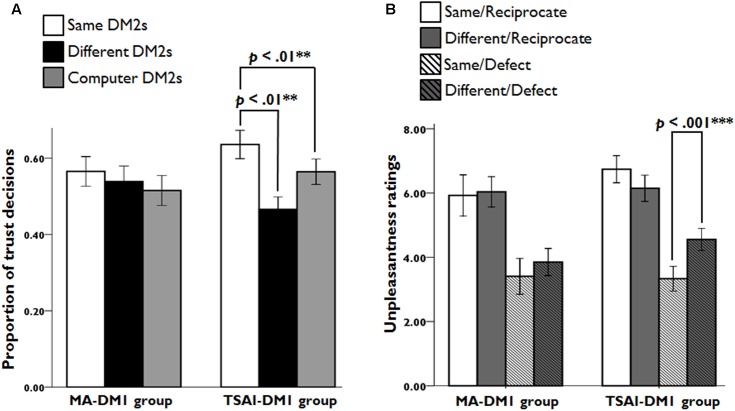
The trust rates and unpleasantness ratings for each condition. **(A)** Participants of the TSAI-DM1 group chose to trust SAME-DM2s more often than DIFF-DM2s and COM-DM2s. **(B)** Based on the questionnaires after the fMRI recording sessions, participants of the TSAI-DM1 group had greater feelings of unpleasantness in Same/Defect trials relative to Different/Defect trials.

We analyzed the percentage of trust decision for each DM1-GROUP and DM2-TYPE across the entire trust game. There was a significant interaction of DM1-GROUP × DM2-TYPE (*F*_2,104_ = 4.63, *p* = 0.012, η^2^ = 0.076), but no significant main effects of DM2-TYPE and DM1-GROUP. Notably, further analysis revealed that TSAI-DM1s made more trust choices when they were playing with SAME-DM2s than when they were playing with DIFF-DM2s (*t*_26_ = 3.18, *p* = 0.004, *d* = 0.87) and with COM-DM2s (*t*_26_ = 2.82, *p* = 0.009, *d* = 0.57). However, no such differences were observed for MA-DM1 group members. These findings revealed that the averaged behavior response pattern had different characteristics for the two DM1 groups: TSAI-DM1 group generally has higher trust to in-group DM2 or COM-DM2 as compared to out-group DM2, whereas MA-DM1 group treats equally to all three types of DM2 players (**Figure 2A**).

An ANOVA on the unpleasantness ratings to the DM2s’ negative returns, with DM1-GROUP and DM2-TYPE, revealed a significant main effect of DM2-TYPE (*F*_1,52_ = 19.24, *p* < 0.001, η^2^ = 0.27), but not of DM1-GROUP. An interaction of DM1-GROUP and DM2-TYPE was observed (*F*_1,52_ = 4.19, *p* = 0.046, η^2^ = 0.075). A *post hoc* analysis showed that TSAI-DM1s had more feelings of unpleasantness during interactions with defected SAME-DM2s (*t*_26_ = -4.64, *p* < 0.001, *d* = 1.29). However, there was no such differences for MA-DM1 group members (*t*_26_ = -1.61, *p* = 0.12, *d* = 0.34). These suggest that TSAI-DM1s’ resentment was particularly high when they received a negative return from the in-group DM2s. For the unpleasantness ratings to DM2s’ positive returns, a mixed ANOVA showed no significant main effect of DM1-GROUP and DM2-TYPE, and no significant two-way interaction of DM1-GROUP × DM2-TYPE (**Figure 2B**).

### Brain Responses (OUTCOME Phase) to Different Partners (DM2-TYPE)

Even though participants were informed that they were playing the trust games with computerized agents who only differ with respect to their identity representation, their brains respond differently between playing a humanoid DM2 (SAME-DM2 and DIFF-DM2) and a computer DM2 (COM-DM2). First, we observed a significant main effect of DM2-TYPE in bilateral TPJ, bilateral caudate, bilateral middle frontal gyrus (MFG), bilateral middle temporal gyrus (MTG), bilateral DLPFC, bilateral occipitotemporal region, right posterior cingulate cortex (PCC), right ACC, right superior temporal gyrus (STG), left medial prefrontal cortices (mPFC), and left AI. Please refer to **Table 1** for results from the follow-up analyses. Conjunction analysis further revealed that participants showed overlapping activations in right MFG, bilateral TPJ, bilateral MTG, right DLPFC, and right PCC when they were playing with a humanoid DM2 than when they were playing with a computer DM2 [(SAME-DM2 > COM-DM2) ∩ (DIFF-DM2 > COM-DM2)] (**Table 1**). Most of these regions have been consistently associated with mentalizing processes of other people’s behaviors (Amodio and Frith, 2006; Van Overwalle, 2009). These results are in line with previous findings (Braynov, 2013) that playing trust games with a human-like partner, as opposed to a computer partner, activates a diverse social brain network. We also found significant political-identity-based differences in response to game playing with a partner of the same versus different political identity. Specifically, there was stronger activation in bilateral caudate when facing DM2 of the same identity than when facing DM2 of the different identity (**Table 1**).

**Table 1:** Brain areas activated in response to different partners (DM2-TYPE).

	MNI coordinates				
Brain area	*X*	*y*	*z*	*Z*-score	*K*
SAME-DM2 > COM-DM2					
Middle frontal gyrus	42	16	58	4.59	382
Middle frontal gyrus	-40	18	54	4.57	105
Dorsolateral prefrontal gyrus	16	50	40	4.63	1780
Dorsolateral prefrontal gyrus	-4	62	32	4.95	1780
Temporoparietal junction	-54	-66	30	6.06	2783
Posterior cingulate cortex	2	-50	28	6.13	1046
Temporoparietal junction	56	-68	10	6.38	4055
Middle occipital gyrus	-46	-78	2	6.62	2783
Medial prefrontal cortex	0	52	-14	5.02	108
Anterior insula	-30	18	-14	4.44	153
Superior temporal gyrus	38	-50	-20	4.17	155
DIFF-DM2 > COM-DM2					
Middle frontal gyrus	-40	18	56	5.05	95
Middle frontal gyrus	46	20	50	4.33	151
Dorsolateral prefrontal gyrus	20	44	42	4.25	280
Posterior cingulate cortex	2	-50	28	6.09	771
Temporoparietal junction	-46	-62	28	5.50	994
Temporoparietal junction	58	-64	18	5.89	1551
Middle temporal gyrus	-50	-10	-20	5.13	695
Middle temporal gyrus	54	-2	-24	4.54	281
COM-DM2 > SAME-DM2					
Superior occipital gyrus	-36	-88	22	5.66	72
Occipitotemporal region	-26	-44	-14	5.14	178
COM-DM2 > DIFF-DM2					
Superior occipital gyrus	-34	-88	24	5.95	164
Occipitotemporal region	30	-46	-10	6.44	122
Occipitotemporal region	-30	-44	-10	6.35	109
Lingual gyrus	-26	-62	-16	5.13	1341
SAME-DM2 > DIFF-DM2					
Caudate	-2	12	12	5.09	91
Caudate	6	18	10	3.37	91
DIFF-DM2 > SAME-DM2					
None					
Humanoid-DM2 > non-humanoid-DM2 [(SAME-DM2 > COM-DM2) ∩ (DIFF-DM2 > COM-DM2)]					
Middle frontal gyrus	42	16	58	4.50	154
Dorsolateral prefrontal gyrus	20	46	50	3.55	85
Temporoparietal junction	-46	-64	30	4.90	324
Temporoparietal junction	56	-66	28	5.62	1004
Posterior cingulate cortex	6	-54	28	4.70	402
Non-humanoid-DM2 > humanoid-DM2 [(COM-DM2 > SAME-DM2) ∩ (COM-DM2 > DIFF-DM2)]					
Occipitotemporal region	30	-46	-10	6.19	101
Superior occipital gyrus	-36	-88	22	5.32	161
Occipitotemporal region	-26	-44	-16	5.06	95

*Here we present brain activation associated with the OUTCOME phase. All clusters reached significance at family-wise error (FWE) rate-corrected *p* < 0.05 (voxel-wise uncorrected *p* < 0.001 and a spatial extent threshold > 70)*.

### Brain Responses (OUTCOME Phase) to Different Outcomes (Reciprocation vs. Defection Outcomes)

**FIGURE 3:**
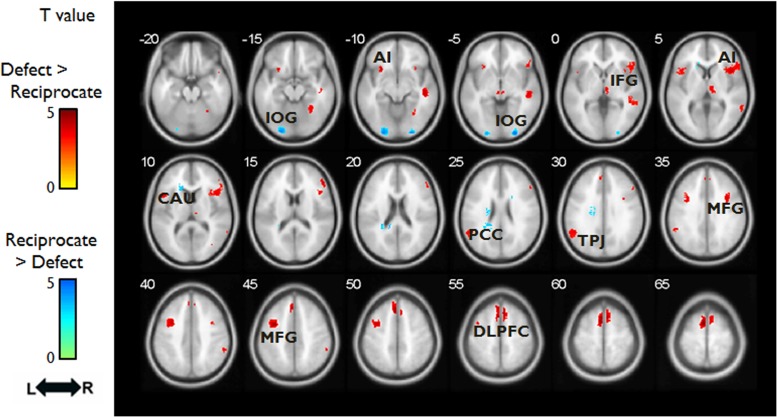
Hemodynamic responses (OUTCOME phase) to different outcomes. Red/yellow colors label the intensity of hemodynamic responses that were higher in the Defect outcomes than the Reciprocate outcomes; whereas the blue/green colors label the intensity of hemodynamic responses that were higher in the Reciprocate outcomes than the Defect outcomes. Abbreviations: DLPFC, dorsolateral prefrontal cortex; MFG, middle frontal gyrus; TPJ, temporoparietal junction; PCC, posterior cingulate cortex; CAU, caudate; AI, anterior insula; IFG, inferior frontal gyrus; IOG, inferior occipital gyrus.

As expected, we observed activation in regions involved in emotional processing associated with the defect outcomes in which partners decide to betray DM1’s trust (Aimone and Houser, 2011; Cloutier et al., 2011; Aimone et al., 2014). Whole brain analysis showed that there was a main effect of OUTCOME in bilateral DLPFC, bilateral MFG, bilateral inferior occipital gyrus, right STG. Follow-up analysis revealed that reciprocate outcomes, relative to defect outcomes [(SR+DR)-(SD+DD)], elicited greater signal changes in left inferior occipital gyrus; whereas defect outcomes, relative to reciprocate outcomes [(SD+DD)-(SR+DR)], elicited more activation in bilateral DLPFC, left MFG, and right STG (**Table 2** and **Figure 3**). There were no group-wise differences in brain response to outcomes.

**Table 2:** Brain areas activated in response to different outcomes.

	MNI coordinates				
Brain area	*x*	*y*	*z*	*Z*-score	*K*
Reciprocate > Defect					
Inferior occipital gyrus	-24	-94	-10	3.86	126
Defect > Reciprocate					
Dorsolateral prefrontal cortex	-4	4	64	4.55	248
Dorsolateral prefrontal cortex	6	14	60	4.14	146
Middle frontal gyrus	-36	4	48	4.16	184
Superior temporal gyrus	48	-24	-6	3.96	102

*Here we present brain activation associated with the OUTCOME phase. All clusters reached significance at family-wise error (FWE) rate-corrected *p* < 0.05 (voxel-wise uncorrected *p* < 0.001 and a spatial extent threshold > 70)*.

### Brain Response (OUTCOME Phase) to Conflicts of Social Expectancy (Three-Way Interaction of DM1–DM2 Pairs by Outcomes)

There is a significant interaction of DM2-TYPE and OUTCOME in bilateral DLPFC, right MTG, and bilateral caudate. Follow-up analysis only revealed that functional contrasts between defect outcomes and reciprocate outcomes in the in-group trials elicited activations in bilateral DLPFC and right MTG, and functional contrasts between reciprocate and defect outcomes in the out-group trials elicited activations in bilateral caudate (**Table 3**).

**Table 3:** Brain areas activated in response to different partner–outcome pairs (interaction of DM2-TYPE × OUTCOME).

	MNI coordinates				
Brain area	*x*	*y*	*z*	*Z*-score	*K*
SAME-DM2 defects > SAME-DM2 reciprocates					
Dorsolateral prefrontal cortex	2	18	58	3.55	131
Dorsolateral prefrontal cortex	-4	10	52	3.26	131
Middle temporal gyrus	46	-42	2	3.71	162
DIFF-DM2 reciprocates > DIFF-DM2 defects					
Caudate	0	12	12	3.63	89
Caudate	-2	20	12	3.42	89

*Here we present brain activation associated with the OUTCOME phase. All clusters reached significance at family-wise error (FWE) rate-corrected *p* < 0.05 (voxel-wise uncorrected *p* < 0.001 and a spatial extent threshold > 70)*.

Our exploratory whole brain analyses (uncorrected *p* < 0.001 and an cluster extent *k* > 10 voxels) for the three-way interaction effect of DM1-GROUP × DM2-TYPE × OUTCOME showed a trend that participants from the two different political identity groups respond differently when faced with conflicts against self-expectation regarding in-group versus out-group trust trials. There was three-way interaction effect of DM1-GROUP, DM2-TYPE, and OUTCOME within bilateral DLPFC, bilateral TPJ, left AI, and left caudate (Supplementary Table S2). Follow-up analyses further revealed that TSAI-DM1 group showed significant higher signal changes than MA-DM1 group, in the functional contrast between SD and SR trials [TSAI-DM1(SD-SR) > MA-DM1(SD-SR)] within bilateral DLPFC, bilateral TPJ, and left AI. In addition, TSAI-DM1s showed greater activation than the MA-DM1s in left caudate in response to DR trials compared with DD trials [TSAI-DM1(DR-SR) > MA-DM1(DR-SR)]. Please note, however, that these three-way interaction effects did not survive the more stringent cluster threshold setting.

### ROI Analyses

**FIGURE 4:**
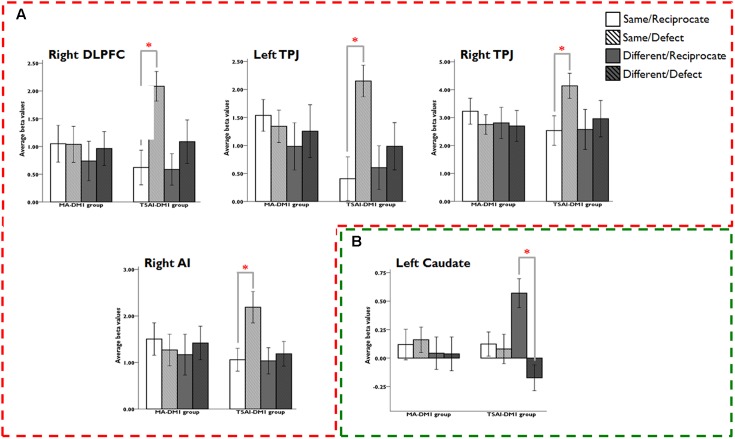
Results of ROI analyses for the three-way interaction (DM1-GROUP × DM2-TYPE × OUTCOME). The ANOVA analyses revealed that: **(A)** only the TSAI-DM1 group showed significant stronger activation for the Same/Reciprocate outcome than the Same/Defect outcome in bilateral DLPFC, bilateral TPJ, and left AI; and **(B)** only the TSAI-DM1 group showed significant stronger activation for the Different/Reciprocate outcome than the Different/Defect outcome in left caudate. Abbreviations: DLPFC, dorsolateral prefrontal cortex; TPJ, temporoparietal junction; AI, anterior insula.

Results from the ROI analyses of the three-way interaction (DM1-GROUP × DM2-TYPE × OUTCOME) revealed that only TSAI-DM1 group showed sensitivity to the in-group versus out-group trust games: activity in DLPFC, TPJ, and AI correlated with negative outcomes in in-group interactions whereas activities in caudate correlated with positive outcomes in out-group interactions. There were significant three-way interactions of DM1-GROUP, DM2-TYPE, and OUTCOME in right DLPFC (*F*_1,52_ = 7.68, *p* = 0.008, η^2^ = 0.13); left TPJ (*F*_1,52_ = 6.27, *p* = 0.015, η^2^ = 0.11), right TPJ (*F*_1,52_ = 4.16, *p* = 0.046, η^2^ = 0.07); right AI (*F*_1,52_ = 7.89, *p* = 0.007, η^2^ = 0.13), and left caudate (*F*_1,52_ = 4.62, *p* = 0.036, η^2^ = 0.08). *Post hoc* analysis revealed that TSAI-DM1 group had greater right DLPFC (*t*_26_ = 6.29, *p* < 0.001, *d* = 0.98), left TPJ (*t*_26_ = 4.00, *p* < 0.001, *d* = 1.00), right TPJ (*t*_26_ = 7.41, *p* < 0.001, *d* = 0.58), and right AI (*t*_26_ = 4.24, *p* < 0.001, *d* = 0.75] activations in response to the SD compared with the SR condition, but not MA-DM1 group (**Figure 4**). Similarly, only TSAI-DM1 group showed a greater signal change in left caudate in response to the DR condition than the DD condition (*t*_26_ = 5.35, *p* < 0.001, *d* = 1.20), and again, no such effect was found in caudate between the DR and DD conditions for the MA-DM1 group (**Figure 4**).

#### Correlation Analyses between ROI-Based Activities and the Unpleasantness Ratings

**FIGURE 5:**
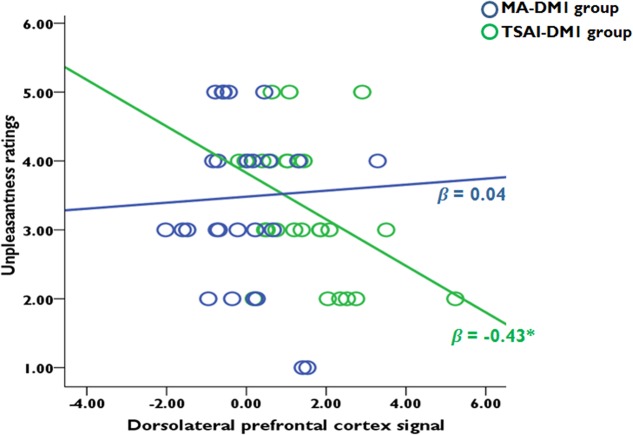
Correlation analyses between ROI-based hemodynamic responses (SAME-Defect vs. SAME-Reciprocate) and subjective unpleasantness ratings when receiving defect outcomes from the in-group partners. There was a negative correlation in the ROI of dorsolateral prefrontal cortex only for the TSAI-DM1 participants, but not for the MA-DM1 participants.

We performed a step-wise regression analysis with strength of activations [beta-values (SD-SR)] in all selected ROIs as predictor variables and subjective unpleasantness ratings (acquired after the fMRI sessions) as a dependent variable. When the two DM1 groups combined, the results did not find any effects from each ROI on subjective unpleasantness of the SD conditions. Within each group, a stepwise multiple regression model revealed that activities in right DLPFC [(10, 12, 52), Gabay et al. (2014)] partially contribute to the subjective unpleasantness of the SD conditions only in TSAI-DM1 group (standardized β = -0.43, *t*_26_ = -2.35, *p* = 0.027), but not for the MA-DM1 group. A Fisher test upon the transformed *r*-values confirmed that this correlation was significantly stronger for participants from the TSAI-DM1 group than those from the MA-DM1 group (*z* = 1.72, *p* < 0.05). Specifically, a stronger DLPFC activation correlated with increased unpleasantness ratings when receiving defect outcomes in the in-group trials (**Figure 5**). Note that the inclusion of TPJ, AI, and caudate did not improve the fitting performance of the model. Furthermore, multiple regression analyses did not find any correlation between unpleasantness ratings for other outcome conditions and hemodynamic responses for these ROIs in both groups.

## Discussion

In the current fMRI study, we demonstrated significant modulatory effects of group identity upon individuals’ behaviors and neural activities associated with reward perception, emotional salience, mentalizing, and cognitive control in trust-related decision making. Interestingly, unlike the general prediction by the social identity theory, groups defined by different political orientation did not show similar degrees of in-group favoritism. First, we observed a behavioral shift toward higher trust toward in-group partners (SAME-DM2s) only in the TASI-DM1 group, but not in the MA-DM1 group. Neuroimaging findings further suggested that only the TSAI-DM1 group showed the sensitivity to outcome conflicts of political orientation-based social expectation in trust games. This effect is also manifested by a positive correlation between neuronal activations in DLPFC and subjective reports of unpleasantness for receiving negative outcomes from in-group games. Since there were no evident differences in the personality traits and the degrees of social capital between the two groups, the possibility that some inherent group difference of individual characteristics may have accounted for the observed group differences of trust behavior patterns can at least be partially ruled out.

The incomparable effects of in-group favoritism between the two identity groups during the trust game seem to imply that the intergroup bias based on political identity cannot be fully explained by the social identity theory and that acquired identities may be functionally different from the assigned identity (Huddy, 2001, 2003). Moreover, given that participants were informed about the 50% overall reciprocate rate across all partner types (DM2), it is reasonable that the trust ratios of MA-DM1s were similar across different partner types and only varied slightly around 50%. Contradictorily, TSAI-DM1s still showed significant in-group favoritism in trust decision making. Our results are in line with previous political studies in Taiwan (Kao and Li, 2000; Yang et al., 2014) which showed that DPP supporters show stronger in-group preferences than KMT supporters when interacting with people having the same political identity. Notably, most of the TSAI-DM1 participants support DPP, which is the major opposition party in Taiwan and closely linked to the initiation of several well-known social and mass movements (Scott, 2006). Due to DPP’s special historical background (DPP emerged from the martial law era of Taiwan), its supporters tend to have strong commitment to each other and trust partisan institutions/agents (Shi, 2014). The current results seemed to match Yamagishi’s observation that in-group favoritism in economic games occurs only when participants believe other in-group members will reciprocate the favor (Yamagishi and Kiyonari, 2000). Furthermore, it also echoes the concerns (Huddy, 2001, 2003) that the variability of identity strength in political orientation might render additional influence on the prediction of social identity theory. For the current fMRI study, all participants were asked again if the presidential election were to occur the next day (which was around 2 years after the 2012 election) whether they would make the same vote as they did before. For the TSAI-DM1 group, all participants would make the same vote as they did in 2012, however, for the MA-DM1 group, only 55% of them would make the same vote as they did in 2012. This provides indirect evidence that the varied identity strength of the MA-DM1 group might be a potential factor that brings about the observed group difference. In addition, although the fMRI data were collected almost 2 years after the election, we cannot completely rule out the possibility that there might be left-over aversive emotion induced by losing the election that may continue to influence participants’ trust behaviors toward outgroup members. However, to provide direct evidence to distinguish between these possibilities, future studies with more sophisticated measures (e.g., impression scales toward ingroup/outgroup members, levels of emotional disturbance induced by ingroup/outgroup members, etc.) will be needed to answer this question.

The unique neuronal activation patterns of the TSAI-DM1 help extend our understanding of group specific effects upon conflicts of social expectances. First, AI has been shown to be involved in subjective experience of emotions and trust evaluations (Phan et al., 2010; Schipper and Petermann, 2015). Recent fMRI research found that enhanced AI activity occurs during the violation of social norms as in unreciprocated cooperation and unfairness (Aimone and Houser, 2011; Guo et al., 2014). Our ROI-based results further showed identity differences can modulate emotion-related brain activity in AI for detecting deviations from social expectancies. Second, in addition to brain regions associated with emotional processing, participants from the TSAI-DM1 group showed significant activations in the regions implicated in effortful causal reasoning (DLPFC) and mental state attribution (TPJ) during conflicts of social expectancy (Bartholow et al., 2006; Cloutier et al., 2011). Furthermore, only the TSAI-DM1 group showed significant negative correlation between DLPFC activation and subjective unpleasantness feelings when receiving a negative return from the in-group partners, suggesting that DLPFC may not only signal and evaluate conflicts of social expectancies, but also help regulate negative emotions that come with the conflicts (Decety and Lamm, 2006). Note that we failed to observe any social expectancy conflict-related activations in ACC, which is known to be involved in extracting trustworthiness information and conflicts monitoring (Platek et al., 2009). The null finding of ACC activation may be due to the fact that we used the compound condition design which has been shown to be less sensitive to conflict-related activity in ACC (Cloutier et al., 2011).

Interestingly, even though participants knew that all partners in the current game were simulated computer agents, we still found enhanced activation within TPJ, mPFC, and PCC when they were playing with “simulated human” partners (SAEM-DM2s and DIFF-DM2) compared to “computer” partners (COM-DM2s). There was evidence showing that human–computer interactions are functionally different from human–human interactions in trust behaviors (McCabe et al., 2001). Therefore, our results seem to suggest that participants were indeed treating the humanoid computer agent as if they were real people. Since investors’ decisions rely profoundly on what they know about the beliefs and intentions of the corresponding trustees, mentalizing is an important process for investors to integrate mutual pay-off information in trust game playing (McCabe et al., 2001; King-Casas et al., 2005; Krueger et al., 2008). Accumulating neuroimaging evidence has suggested that regions in TPJ, mPFC, and PCC play critical roles in social information evaluation, perspective taking, and sense of agency within a context where another individual’s intentions must be taken into account before acting (Van Overwalle, 2009). In trust games, these brain regions may therefore be involved with mentalizing processes to predict a partner’s behavior and determine whether s/he is trustworthy (Fett et al., 2014). Note that given the limitation of the current experimental design, we are unable to claim that these brain regions involved with mentalizing or reward valuation processes exclusively in trust-related interaction. Future studies that compare different types of social interaction will be needed to answer whether there are trust-specific loci in these social cognition brain regions.

## Conclusion

In summary, our behavioral and neuroimaging results converged to demonstrate the robust influence of acquired social identity upon interpersonal trust interactions. Furthermore, the current findings imply that the social characteristics of an identity group may render additional influence upon people’s expectancy toward their partners within an in-group or out-group interaction. At the neural level, distinct brain regions were found to be associated with different expectancy violations: unexpected negative outcomes from in-group members were linked to AI, TPJ, and DLPFC, whereas unexpected rewards from the out-group members were linked to caudate. Furthermore, brain responses in DLPFC correlated with negative emotions arising from in-group defection. This study complements existing literature on the role of social identity in trust behaviors and provides new insights into the brain mechanisms underlying social expectancy violations in different identity-based social contexts.

## Author Contributions

C-TW participated in the study conception, designed the experiment protocols, data analysis and interpretation, and manuscript writing. Y-TF performed the data analysis, data interpretation, and manuscript writing. Y-RD led the data acquisition and performed data analysis. T-TY led the data acquisition. H-LL, N-SY, and S-HC participated in the study conception and experimental designs. R-MH was the primary investigator who initiated the whole project, led the study conception, designed the experiments, led data interpretation, and critically read and commented on the manuscript.

## Conflict of Interest Statement

The authors declare that the research was conducted in the absence of any commercial or financial relationships that could be construed as a potential conflict of interest. The reviewer AP and handling Editor declared their shared affiliation, and the handling Editor states that the process nevertheless met the standards of a fair and objective review.
