# Supplementary material for: How Do Acquired Political Identities Influence Our Neural Processing toward Others within the Context of a Trust Game?
Source: Front Hum Neurosci. 2018 Feb 2;12:23. doi: 10.3389/fnhum.2018.00023 (PMC5801296; doi:10.3389/fnhum.2018.00023)
Supplement: Supplementary file 1 [file Presentation_1.PDF]

Supplementary brief historical background of the two-party system in Taiwan

Taiwan had been a one-party (Kuomingtang, KMT) political regime since Chiang Kai-shek was defeated and retreated to Taiwan in 1949. After the retreat, Chiang Kai-shek soon became the first president and remained in the office until his death in 1975. His son, Chiang Ching-kuo, was subsequently elected as the next president by the Assembly Representatives in 1978 and remained in the office until his death in 1988. In 1987, the lift of martial law, proclaimed by President Chiang Ching-kuo, allowed the establishment of the opposition party - Democratic Progressive Party (DPP). The first democratic election for the president by all citizens in Taiwan was held in 1996, which was the starting point of the long lasting competition for the presidential seat between KMT and DPP. For the past few decades, the opinions on the issues of Taiwan’s independence and national identity during elections have diverged between KMT and DPP Parties. The tension between supporters of the two parties was notoriously high during important elections (e.g., president, mayors, legislators), most likely induced by opposition on the issue of Taiwan's independence during the presidential elections. The major divergence between two parties in the presidential election of 2012 was mainly on the issue of national identity. KMT party supporters favor the 1992 consensus on national identity (i.e., one China and two political entities or one China and respective interpretation) whereas the DPP party supporters favor Taiwan identity and Taiwan’s Independence (Cheng 2013, Liu & Li 2017). Surprisingly, the political identity on one China or Taiwan independence even influenced in the trust exchanges of the interactions to strangers in daily life (Yang et al., 2014).

References

Cheng, S-F. (2013). Identity in the 2012 Taiwanese presidential election. *Issues & Studies, 52*(4), 101-132.

Liu, F. C. S., & Li, Y. (2017). Generation matters: Taiwan's perceptions of mainland China and attitudes towards Cross-Strait trade talks. *J Contemp China*, *26*(104), 263-279.

Yang, T.-T., Hsung, R.-M., Chen, S.-H., Du, Y.-R., & Lin, Y.-J. (2014). *Mechanisms of trust formation under different conditions of political identity*. Paper presented at the XVIII ISA World Congress of Sociology, Yokohama, Japan. (https://isaconf.confex.com/isaconf/wc2014/webprogram/Paper48095.html)

Supplementary task instruction for participants

For this fMRI experiment, you will perform a total of 6 sessions of trust games with each session contains 36 rounds. For each round, the game partner you will be facing will be randomly selected from one of the following computer agents displayed as: 1. a computer screen image, 2. a face image covered by a blue oval (representing someone who voted for Ma Ying-Jeou in 2012), 3. a face image covered by a green oval (representing someone who voted for Tsai Ing-Wen in 2012). In the beginning of each round, you will be provided with a starting fund of 20 monetary units (MU) and you can decide whether to hold [KEEP] the 20 MU or invest [TRUST] the 20 MU to the other player agent. Please press the left key for the KEEP and the right key for the TRUST. The other player agent can respond with either reciprocate your trust [RECIPROCATE] or defect your trust [DEFECT]. Table S1 lists the payoff results for each decision scenario. If you decide to hold the investment (KEEP) for a given round, the round will be finished by the starting fund being equally distributed between you and the other player: you will receive 10 MU and the other player will receive 10 MU. However, you will still see the response (RECIPROCATE or DEFECT) from the other player for that round (after you make the KEEP decision). If you decide to invest (TRUST), the starting fund will be doubled to be 40 MU and there will be two possible scenarios: 1. you will receive 20 MU if the other player choose to reciprocate your trust (RECIPROCATE) or 2. you will receive 0 MU if the other player choose to defect your trust (DEFECT). Please note that, all in all, there will be half of the player agents who reciprocate your trust and half of them who defect your trust.

Table S1 Payoff tables

	You choose TRUST<br><br>The other player choose RECIPROCATE	You choose TRUST<br><br>The other player choose DEFECT	You choose KEEP<br><br>The other player choose RECIPROCATE	You choose KEEP<br><br>The other player choose DEFECT
Outcomes	20	0	20	0
(fixation point)	•	•	•	•
Amount you receive	20	0	10	10

Table S2 Brain areas activated in response to different combination of participant-partner-outcome from exploratory analyses (interaction of DM1-GROUP × DM2-TYPE × OUTCOME)

Brain area	MNI Coordinates x	y	z	*Z* score	*k*
TSAI-DM1 (SAME-DM2 defects - SAME-DM2 reciprocates) > MA-DM1 (SAME-DM2 defects - SAME-DM2 reciprocates)					
Dorsolateral prefrontal cortex	2	14	60	3.46	32
Dorsolateral prefrontal cortex	–4	10	56	3.34	28
Temporoparietal junction	58	–48	46	3.22	22
Temporoparietal junction	–52	–50	30	3.28	26
Anterior insula	–28	18	–12	3.27	15
MA-DM1 (SAME-DM2 defects - SAME-DM2 reciprocates) > TSAI-DM1 (SAME-DM2 defects - SAME-DM2 reciprocates)					
None					
TSAI-DM1 (DIFF-DM2 reciprocates - DIFF-DM2 defects) > MA-DM1 (DIFF-DM2 reciprocates - DIFF-DM2 defects)					
Caudate	–2	20	12	3.36	18
MA-DM1 (DIFF-DM2 reciprocates - DIFF-DM2 defects) > TSAI-DM1 (DIFF-DM2 reciprocates - DIFF-DM2 defects)					
None					

All clusters reached significance at voxel-wise uncorrected $p < .001$, a spatial extent threshold > 10.
